# Exploration of Key Flavor Compounds in Five Grilled Salmonid Species by Integrating Volatile Profiling and Sensory Evaluation

**DOI:** 10.3390/metabo16010030

**Published:** 2025-12-26

**Authors:** Yuka Mori, Akimasa Hatanaka, Eiichiro Fukusaki

**Affiliations:** 1 Department of Biotechnology, Graduate School of Engineering, The University of Osaka, 2-1 Yamadaoka, Suita, Osaka 565-0871, Japan; yuka_mori@nissui.co.jp; 2 Central Research Laboratory, Nissui Corporation, 1-32-3 Nanakuni, Hachioji-shi, Tokyo 192-0991, Japan; hatanaka@nissui.co.jp; 3 Osaka University Shimadzu Omics Innovation Research Laboratories, The University of Osaka, 2-1 Yamadaoka, Suita, Osaka 565-0871, Japan

**Keywords:** grilled salmonid species, sensory evaluation, GC/MS, GC/O, OPLSR, additive test

## Abstract

**Background/Objectives**: Salmonid species are globally popular and widely consumed in Japan, especially when grilled. Understanding their flavor characteristics from sensory and compositional perspectives is essential to improve the quality of processed salmonid products. However, scientific knowledge in this area remains limited. This study aimed to explore compounds contributing delicious flavor of grilled salmon by performing correlation analysis between sensory evaluation and volatile profiles of five grilled salmonid species. **Methods**: To characterize each sample, sensory evaluation using paired comparisons and comprehensive analysis of volatile compounds by gas chromatography/mass spectrometry (GC/MS) were conducted. To select compounds strongly associated with the “delicious flavor of grilled salmon”, orthogonal partial least squares regression (OPLSR) and gas chromatography/olfactometry (GC/O) were performed. A subset of the selected candidate compounds was quantified, and additive tests on the samples were carried out based on their concentrations. **Results**: Sensory evaluation revealed clear differences in flavor profiles among the five salmonid species. A total of 344 peaks were detected in all samples, and principal component analysis (PCA) of these data showed grouping trends consistent with those obtained from sensory evaluation. OPLSR using sensory and volatile data as variables, and GC/O analysis identified 23 compounds, including trimethylamine, dimethyl sulfide, and 1-heptanol, as candidates contributing to the characteristic flavor of grilled salmonid species. Sensory evaluation of samples supplemented with a subset of these candidates showed that, particularly at the higher addition level, aroma and flavor tended to approach those of the highly preferred samples. **Conclusions**: These findings suggest that some of the selected candidate compounds contribute to the formation of delicious flavor of grilled salmon and may be useful for flavor design and quality improvement of processed products made from salmonid species.

## 1. Introduction

Salmonids are popular seafood products that are in high demand worldwide. According to the latest statistics reported by the Food and Agriculture Organization of the United Nations (FAO, 2024), finfish accounted for 65% of the total global trade value of aquatic animal products in 2022, with salmonid species comprising approximately one-third of this category and thus representing the most traded finfish group [1]. Japan has a high demand for salmonids. According to statistics from the Ministry of Agriculture, Forestry, and Fisheries (MAFF), salmonid species ranked first in the annual per capita purchase volume of fresh seafood during the most recent reporting period from 2004 to 2023. In recent years, advances in production technologies and distribution systems have led to salmonids being marketed not only as fresh products but also widely as various processed products, including canned, smoked, fried, and marinated products. These products provide convenience and extended shelf-life by removing inedible portions and applying seasoning and heat treatment, making them an important supply form.

However, external factors such as temperature fluctuations and oxidation during distribution and storage of processed foods may lead to degradation or loss of the volatile compounds that characterize product flavor. For example, Strecker aldehydes, which contribute to the desirable roasted flavor of cooked chicken patties, decrease during refrigerated storage and reheating [2]. The volatile compounds that contribute to the spicy and meaty flavors of cooked beef meatballs are similarly reduced during frozen storage [3]. Among seafood products, levels of aldehydes associated with sweetness and a nutty flavor, as well as the esters decrease during storage at room temperature [4]. These changes in flavor compounds might not only affect consumer palatability but also decrease the product value and market competitiveness. Therefore, scientifically identifying the key flavor compounds that characterize the flavor of processed products and establishing processing technologies to maintain or supplement these compounds are critical challenges for the development of seafood products. Salmonid species are commonly grilled in Japan, and elucidating their characteristic flavor compounds has significant academic and industrial relevance. Several characteristic flavor compounds have been reported in salmon products processed by steaming, pressure cooking, or smoking. For example, aldehydes such as hexanal and alcohols such as (E)-2-octenol are key contributors to the flavors of steamed Atlantic salmon [5]. Sulfur-containing compounds such as dimethyl disulfide contribute to the development of a characteristic flavor in cooked canned salmon products when combined with carbonyl compounds, such as 2-methylbutanal and 2,3-pentanedione [6,7]. Phenolic and furan compounds are the major contributors to the flavor of smoked salmon [8,9]. However, studies on flavor compounds in grilled salmonids remain scarce, and scientific knowledge is limited. Aldehydes and ketones might be characteristic of oven-heated salmon [10]. The flavors of *trans*, *cis*-2,6-nonadienal, 2-hexenal, and 2,3,5-trimethylpyrazine were prominent in pre- and post-cooking flavor comparisons of three salmon varieties [11]. However, the key compounds in these species have not been comprehensively identified by integrated sensory evaluation and volatile profiles in several salmonid species that are popular in Japan. Moreover, the influence of these compounds on human sensory perceptions or preferences is unknown.

This study aimed to explore key flavor compounds that characterize the desirable flavor of grilled salmonid species commonly consumed in Japan by integrating sensory evaluation, volatile profiling, and gas chromatography/olfactometry (GC/O) analysis. Furthermore, we assessed the impact of selected compounds on human perception by adding a subset of these candidates to actual grilled salmonid samples. To the best of our knowledge, this is the first study to identify preference-related volatile compounds that characterize the desirable flavor of grilled salmonid species, based on correlations between volatile profiling and sensory evaluation. These findings may provide a technical foundation for flavor design and raw material selection for processed salmonid products.

## 2. Materials and Methods

### 2.1. Materials

Five grilled salmonid species were prepared and analyzed, including four that belonged to the genus Oncorhynchus: sockeye salmon (*O. nerka*; Japanese name: Benizake; abbreviated as SO), chum salmon (*O. keta*; Japanese name: Shirozake; CH), coho salmon (*O. kisutch*; Japanese name: Ginzake; CO), and rainbow trout (*O. mykiss*). Two types of rainbow trout were used: one reared in salt water (rainbow trout A; RA; Japanese name: Salmon-trout) and another in freshwater (rainbow trout B; RB; Japanese name: Nijimasu). All samples were obtained from local markets. Detailed information on the origin and basic characteristics of each sample is provided in Table S1. For all species except RB, filets were cut, and composite samples were prepared by collecting portions from the anterior (head side), posterior (tail side), central, dorsal, and ventral regions to minimize positional variation within each fish. Because RB individuals were smaller and provided less edible tissue per fish than the other four species, composite samples were prepared from whole fish (after removal of the viscera and head) using the same approach. In all species, the head region (head meat and the area around the gills) was excluded from sampling. All samples, with skin and bones intact and no seasoning applied, were cooked in a preheated oven at 250 °C until the internal temperature exceeded 75 °C and a cooking yield reached 82–85%. The grilled samples were rapidly frozen in vacuum-sealed pouches and stored at −30 °C until analysis.

### 2.2. Reagents

Volatile compounds were quantified using the following reagents: acetaldehyde, dimethyl sulfide, 1-heptanol, ethanol, methanol, hydrochloric acid, and sodium hydroxide (Fujifilm Wako Pure Chemical Co., Osaka, Japan); trimethylamine (TMA), propanal, 2-methylpropanal, 3-pentanone, 1-propanol, S-methyl thioacetate, cyclohexanone, *cis*-3-hexen-1-ol, 2-nonanone, *trans*,*trans*-2,4-heptadienal, 2,6,10,14-tetramethylpentadecane, and propylamine hydrochloride (Tokyo Chemical Industry Co., Ltd., Tokyo, Japan); 2,3-pentanedione (Thermo Fisher Scientific Inc., Waltham, MA, USA); and *trans*-3-hexen-1-ol (Sigma-Aldrich Corp., St. Louis, MO, USA). A domestic flavor manufacturer supplied the food-grade compounds used in the additive tests.

### 2.3. Sensory Evaluation

The quality of the grilled salmonid species was determined by sensory evaluation of paired comparisons with 54 panelists (13 males, 41 females, 24–59 years old) from the Central Research Laboratory of Nissui Corporation. All panelists were informed of the purpose of the study in advance and voluntarily agreed to participate. They were also informed that participation was voluntary and could be withdrawn at any time without any penalty. The samples used in this study were cooked grilled fish commonly consumed in Japan and did not involve any medical procedures, collection of biological specimens, or acquisition of personally identifiable information. Therefore, according to the regulations of the affiliated institution, ethical approval was not required for this study.

Samples for evaluation (as described in Section 2.1) were thawed in a water bath for 15 min. After thawing, the samples were cooled to room temperature (approximately 25 °C), and bones and skin were removed. The flesh was flaked and used as sensory evaluation samples. Operational definitions of the 14 sensory attributes used in the evaluation are summarized in Table S2. Panelists underwent multiple training sessions to understand the definitions of sensory attributes listed in Table S2 and to ensure consistency in terminology interpretation among evaluators. In addition, a preliminary test was conducted prior to the main evaluation to confirm that panelists could reliably distinguish flavor differences among species. Specifically, pairs of randomly selected species from the five were presented, and panelists were asked to choose which sample exhibited stronger intensity for each sensory attribute listed in Table S2. Repeated assessments confirmed the reliability and consistency of panelists’ evaluations.

Two types of shredded grilled salmonid samples were randomly presented to the panelists, who selected the one with the stronger intensity for each sensory attribute (Table S2). To express differences in sensory attributes between samples quantitatively rather than by rank, a scaling method was applied. In this study, several sensory attributes were unanimously selected by all panelists for one of the paired samples. Therefore, Gulliksen’s method, a scaling technique applicable to incomplete paired comparisons data, was employed [12]. Specifically, for each pair, a relative frequency matrix was constructed based on the proportion of panelists who judged one sample as stronger than the other. These proportions were then converted into normalized values (Z-scores) using the inverse cumulative distribution function of the normal distribution. Subsequently, scale values were calculated through matrix operations based on Gulliksen’s algorithm. The resulting scale values were mean centered so that the overall mean was zero.

### 2.4. Analysis of Volatile Compounds

Grilled salmonid species were flaked as described in Section 2.3, mixed with powdered dry ice, and rapidly frozen. The frozen flakes were pulverized with dry ice using a Frestent FST-4000 (AiSTI SCIENCE Co., Ltd., Wakayama, Japan). The powdered samples were stored overnight at −30 °C to sublimate the dry ice. The resulting powder was used for volatile compound analysis. To confirm that the pretreatment did not cause any loss of volatile compounds, the aroma was assessed before and after freeze-grinding and sublimation of dry ice, and no significant changes in aroma quality or intensity were observed. The volatile compounds were extracted using an optimized headspace solid-phase microextraction (SPME) method.

#### 2.4.1. Detection and Identification of Volatile Compounds Using GC/Mass Spectrometry (GC/MS)

Powdered samples (1.0 g) were placed in 20 mL headspace vials and equilibrated at 60 °C for 15 min. A 50/30 μm, 2 cm divinylbenzene/carboxen/polydimethylsiloxane (DVB/CAR/PDMS) fiber assembly (Supelco, Bellefonte, PA, USA) was then inserted into the vial, and volatile compounds were extracted at 60 °C for 30 min. The extracted compounds were thermally desorbed using a GC injection port at 250 °C for 2 min. A 7890A gas chromatograph coupled to a 5975C mass spectrometer (both from Agilent Technologies, Santa Clara, CA, USA) and equipped with a Gerstel Multipurpose Sampler (MPS2XL, Gerstel GmbH & Co., Mülheim an der Ruhr, Germany) was used. Compounds were separated using an InertCap FFAP column (60 m × 0.32 mm × 0.50 µm, GL Sciences Inc., Tokyo, Japan). Pure helium was the carrier gas, and compounds were injected in splitless mode. The GC oven temperature was programmed as follows: held at 40 °C for 3 min, heated from 40 °C to 250 °C at 3 °C/min, and then held at 250 °C for 10 min. The ionization energy was set to 70 eV. The transfer line, ion source, and quadrupole temperatures were 250 °C, 230 °C, and 150 °C, respectively. Mass spectra were recorded over an m/z range of 24–350. All samples were analyzed in technical triplicate to ensure methodological reliability. A pooled quality control (QC) sample was also analyzed under the same conditions. To calculate the retention index (RI), a Qualitative Retention Time Index Standard in hexane (C7–C33, n-alkanes; Restek Corp., Bellefonte, PA, USA) was analyzed under the same conditions. The GC/MS data were first converted to the netCDF format using ChemStation (Agilent Technologies, Santa Clara, CA, USA) and then to the ABF format using an Abf Converter (Reifycs Inc., Tokyo, Japan). The data were subsequently processed using MS-DIAL (v.4.9.221218; RIKEN, Saitama, Japan) for baseline correction, noise removal, peak detection, locally weighted scatterplot smoothing correction, and alignment [13]. Compounds were annotated by comparing the mass spectra with the NIST11 database (NIST, Gaithersburg, MD, USA) and their RI with the Aroma Office database (v.8, Nishikawa Keisoku Co., Ltd., Tokyo, Japan). Where available, the RI and mass spectra of authentic standards analyzed under identical conditions were also used for confirmation. In the NIST11 library search, the EI similarity threshold was set to ≥70, and the RI tolerance was set to ±20. Peaks confirmed using authentic standards were designated as identified, those confirmed based on mass spectra and/or RI were designated as tentatively identified, and all remaining peaks were classified as unknown. Peaks with a relative standard deviation exceeding 30% in the QC sample were excluded from further analysis.

#### 2.4.2. GC/O Analysis

GC/O analysis was performed using the GC/MS system as described in Section 2.4.1, equipped with an olfactometry detection port (ODP2; Gerstel GmbH & Co., Mülheim an der Ruhr, Germany). The carrier gas (pure helium) was used under constant inlet pressure conditions. The column type, oven temperature program, and volatile compound extraction method were described in Section 2.4.1. Volatile compounds that desorbed from the SPME fiber were split between the MS and the sniffing port at a 1:1 ratio. The sniffing port was maintained at 250 °C to prevent the retention of aromatic compounds. The odor intensity and characteristics perceived through the sniffing port were analyzed using a four-point scale (1, weak; 2, moderate; 3, strong; 4, very strong). Humidified air, passed through distilled water, was supplied to the sniffing port at a flow rate of 50 mL/min to prevent nasal dryness in panelists. Each sample was analyzed in triplicate by performing three independent GC injections, and the same trained panelist evaluated the detected odors in all injections to ensure repeatability.

### 2.5. Multivariate Analysis

Volatile compound and sensory evaluation data were analyzed using principal component analysis (PCA) with R software (v.4.2.2) to visualize the overall data structure. Sensory attributes associated with delicious flavor of grilled salmon and volatile compounds correlated with sensory evaluation data were identified using orthogonal partial least squares regression (OPLSR) analysis. OPLSR models were analyzed using SIMCA-P software (v.13, Umetrics, Umea, Sweden). The OPLSR models provided variable importance in the projection (VIP) values and coefficients. Model performance was evaluated based on R^2^ as an index of goodness of fit, Q^2^ as an index of model robustness based on cross-validation, and the root mean square error of estimation (RMSEE). The volatile compound and sensory evaluation datasets were normalized prior to all multivariate analyses using Z-score transformation (mean = 0; variance = 1).

### 2.6. Quantification of Key Flavor Candidates

Among the 23 key flavor candidate compounds identified as potentially important contributors to the flavor of grilled salmonid species based on OPLSR and GC/O analyses, the concentrations of 16 compounds for which authentic standards were available were measured in SO and CO samples. All compounds were analyzed in technical triplicate (*n* = 3), and the results are presented as mean ± standard deviation.

#### 2.6.1. Quantification of Key Flavor Candidates Other than TMA

Except for TMA, the remaining 15 compounds were quantified using the standard addition method described previously [14]. Grilled fish is a complex matrix rich in lipids and proteins, which can strongly influence the extraction efficiency of volatile compounds. Therefore, the standard addition method was adopted, in which authentic standards were directly added to the sample, to minimize matrix effects.

Powdered samples (1.0 g), prepared as described in Section 2.4, and 10 μL of standard were placed in 20 mL headspace vials. Each standard compound was tested at three different concentration levels, including a blank solution (ethanol or methanol). Table S3 shows the concentration range of each compound. The analytical instruments and GC column used were the same as those described in Section 2.4.1. The oven temperature was programmed as follows: held at 40 °C for 3 min, heated from 40 °C to 220 °C at a rate of 3 °C/min, then held at 220 °C for 5 min. Volatile compounds were analyzed by measuring peak areas in selected ion monitoring (SIM) mode. Table S3 shows the target *m*/*z* values used for SIM.

#### 2.6.2. Quantification of TMA

Quantification of TMA was performed using the internal standard method described previously [15,16], with minor modifications. TMA is a highly volatile amine whose transfer behavior to the gas phase varies with pH. Because the relationship between the amount added and the amount recovered tends to be unstable when using the standard addition method, TMA was quantified in this study using an internal standard method.

Calibration curves for TMA were constructed using five concentration levels in the range of 0–7 µg/mL in the headspace vial. Powdered samples (30 g), prepared as described in Section 2.4, were vortex mixed with 20 mL of 0.5 N HCl in plastic centrifuge tubes for 5 min. This acidic condition stabilized TMA in the sample as an ionic amine in the aqueous phase. The mixture was then centrifuged at 3350× *g* for 10 min at 4 °C. Supernatants were passed through No. 5A filter papers (ADVANTEC, Tokyo, Japan) into 50 mL volumetric flasks and brought to volume with 0.5 N HCl to obtain sample solutions. Aliquots (0.5 mL) of sample solutions, 15 M NaOH (1 mL), and an internal standard solution of propylamine hydrochloride (5.0 µg/mL) sealed in 20 mL headspace vials were shaken for 30 s. This alkaline condition converted ionic amines into non-ionic volatile amines and promoted their transfer to the headspace. Samples were equilibrated at 35 °C for 15 min, then DVB/CAR/PDMS SPME fibers were inserted into the vials for extraction at 35 °C for 15 min. Thereafter, thermal desorption was performed in the GC inlet at 200 °C for 5 min. Compounds were separated using GC/MS, as described in Section 2.4.1, with a 60 m × 0.32 mm CP-Volamine capillary column (Agilent Technologies, Santa Clara, CA, USA). The GC oven temperature was programmed as follows: held at 35 °C for 3 min, heated from 35 °C to 200 °C at a rate of 40 °C/min, and then held at 200 °C for 2 min. The analysis proceeded in SIM mode. The target ions were 58 and 59, respectively, for TMA and propylamine hydrochloride.

### 2.7. Additive Tests

Among the 16 compounds quantified in Section 2.6, 9 key flavor candidate compounds were selected for the additive test. These compounds were approved for use in foods in Japan, available in food-grade quality, and present at higher concentrations in SO than in CO. This test was conducted to examine the sensory impact of these selected compounds, which had been identified as potentially important contributors to the characteristic and desirable flavor of grilled salmonid species. The Ethics Committee at Osaka University approved sensory evaluations of ranking tests by 16 panelists (6 males, 10 females, 21–49 years old) from the Graduate School of Engineering at Osaka University (Approval Number Gen-2-1). Prior to the test, the panelists received training to enhance their ability to discriminate flavor differences among grilled salmonid species. This part of the study also complied with the ethical principles enshrined in the Declaration of Helsinki (2013 amendment). All participants were informed of the purpose of the study and voluntarily provided written informed consent to participate. They were also explicitly informed that they could withdraw from the study at any time, without providing a reason.

SO and CO samples were prepared in flaked form as described in Section 2.3. Based on the quantified findings of the key flavor candidates, two test conditions were prepared: one by adding the difference in compound concentrations between SO and CO to the CO sample (Test A: TA), and the other by adding 4-fold this difference to the CO sample (Test B: TB). The 4-fold level was determined based on preliminary tests, which confirmed that higher additions caused noticeable sensory distortion. This setting was chosen to balance flavor enhancement with practical acceptability. Four samples, TA, TB, SO, and CO, were randomly presented to the panelists who ranked them in order of perceived intensity of a pleasant aroma of grilled salmon when smelled and tasted. The panelists wore light-shielding glasses during the evaluation to eliminate visual bias among variable sample colors. The resulting ranks were analyzed using the Friedman rank test, as described previously [17]. Significant differences among samples were further analyzed by calculating rank sum differences (ΔRS) and comparing them to the least significant difference (LSD) calculated based on the number of panelists and samples. Corresponding Z-values were derived using the Ryan method and the NORMSINV function in Microsoft Excel.

## 3. Results

### 3.1. Sensory Characteristics of Grilled Salmonid Species

**Figure 1:**
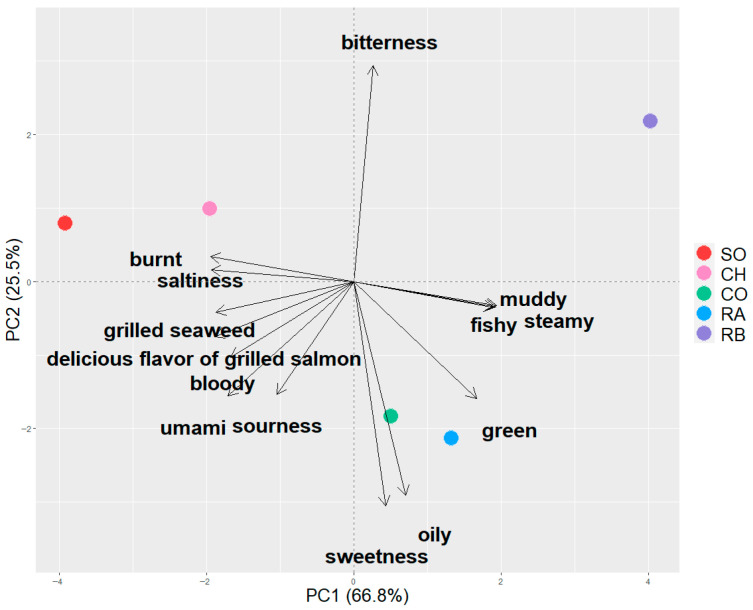
PCA biplot of scores and factor loadings based on sensory evaluation results for 14 sensory attributes of five grilled salmonid species (SO, CH, CO, RA, RB).

Table S4 shows scale values for 14 sensory attributes describing the flavor characteristics of the grilled salmonid species. Figure 1 illustrates the PCA-based visualization of sensory differences among salmonid species. The first and second principal components (PC1 and PC2) together explained 92.3% of the total variance (PC1: 66.8%, PC2: 25.5%), indicating that PCA captured the sensory characteristics of the samples. A biplot revealed that the five samples were broadly classified into three groups. The first group (SO and CH) was plotted in the second quadrant and characterized by high ratings for a delicious flavor of grilled salmon. This group also exhibited strong intensities of “roasted flavor”, “grilled seaweed flavor”, and “saltiness”. The second group (RA and CO), plotted in the fourth quadrant, was characterized by “sweetness”, “oily”, and “green flavor”. The third group, containing only RB, was plotted in the first quadrant and characterized as “muddy flavor”, “steamy flavor”, “fishy”, and “bitterness”. Only RB among the five samples was a freshwater fish. Freshwater fish tend to have stronger “fresh” and “earthy or muddy” odors than marine fish, and the trend was the same in our sensory evaluation [18,19].

The objective of this analysis was to identify the most palatable flavors that characterize grilled salmonid species, specifically, the components most closely associated with overall preferences. In this study, the sensory attribute “delicious flavor of grilled salmon” was used as an indicator of overall preference for the grilled salmonid samples. Accordingly, OPLSR was performed, with the scale values of “delicious flavor of grilled salmon” as the response variable and those of the remaining sensory attributes as explanatory variables. The constructed model was evaluated using R^2^ as an index of goodness of fit, Q^2^ as an index of model robustness based on cross-validation, and RMSEE. The performance of the model is shown in Figure S1. The coefficients were R^2^ = 0.999, Q^2^ = 0.993, and RMSEE = 0.086, indicating an excellent statistical fit compared with commonly used criteria (R^2^ ≥ 0.65, Q^2^ ≥ 0.5, and smaller RMSEE values indicating better performance) [20,21]. However, because the number of samples in this study was small (*n* = 5), R^2^ and Q^2^ values are prone to appear high, and any interpretation of the model’s predictive capability must be made with caution. Therefore, in this study, the model was regarded not as a general predictive model but as an exploratory tool to extract sensory attributes highly associated with the overall evaluation, and the resulting findings were interpreted in conjunction with subsequent analyses.

Subsequently, the sensory attributes that were the most closely associated with the response variable were extracted based on VIP and coefficient values that indicated the degree and direction of contribution to the model (Table 1). Attributes with VIP values greater than 1.1 were considered important contributors. Seven attributes met this criterion: “grilled seaweed flavor”, “muddy flavor”, “steamy flavor”, “roasted flavor”, “fishy”, “saltiness”, and “umami.” Among these, “grilled seaweed flavor”, “umami,” “roasted flavor”, and “saltiness” positively correlated with delicious flavor of grilled salmon. These results showed that the intensities of these four attributes were positively associated with the “delicious flavor of grilled salmon” score. Based on this finding, subsequent analyses focused on these four positively correlated attributes.

In aquatic products, differences in fish species and heat-processing conditions have been reported to manifest as variations in volatile compound profiles, which can be used to classify flavor characteristics [22,23]. Therefore, in this study, we also analyzed volatile compound profiles to capture differences in the four sensory attributes identified among the five salmonid species.

### 3.2. Volatile Profiles of Grilled Salmonid Species

**Figure 2:**
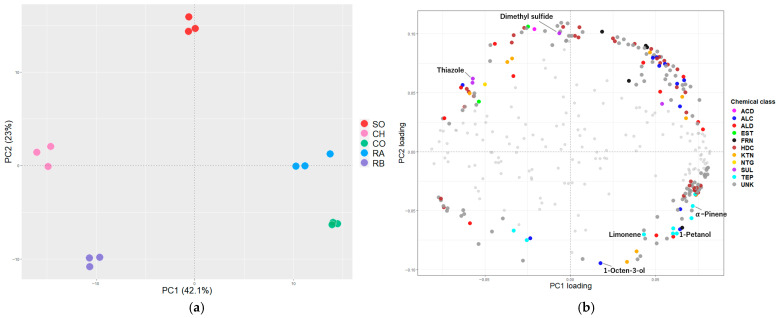
(**a**) PCA score plot of five grilled salmonid species (SO, CH, CO, RA, RB) based on 344 compounds detected by GC/MS; (**b**) PCA loading plot. The top 200 compounds, selected according to the highest cumulative absolute loadings on PC1 and PC2, are color-coded by chemical class (chemical class abbreviation: ACD, acids; ALC, alcohols; ALD, aldehydes; EST, esters; FRN, furans; HDC, hydrocarbons; KTN, ketones; NTG, nitrogen-containing compounds; SUL: sulfur-containing compounds; TEP, terpenes; UNK, unknown).

A total of 344 peaks were detected in grilled salmonid species using non-targeted GC/MS. A complete list of these compounds, including RI, identification methods, and GC/O results, is provided in Table S5. Among these, 141 compounds comprising hydrocarbons, aldehydes, alcohols, ketones, sulfur-containing compounds, and other compounds were identified or tentatively identified. Figure 2a shows the PCA results of all 344 compounds, including 203 unidentified compounds. PC1 and PC2 accounted for 65.1% of the total variance (PC1, 42.1%; PC2, 23.0%). Three distinct groups were observed: SO and CH, RB alone, and CO and RA, which were plotted in the second, third, and near the fourth quadrants, respectively.

This classification was consistent with the PCA results of the sensory evaluation data presented in Section 3.1, suggesting that each species possesses a unique composition of volatile compounds that systematically contributes to its sensory attributes.

To explore volatile compounds contributing to the separation observed in the PCA.

Score plot, the loading plot was examined (Figure 2b). In the positive direction of PC1 (CO and RA), terpenes such as limonene and α-pinene, as well as alcohols including 1-pentanol and 1-octen-3-ol, were located. In contrast, the positive direction of PC2 (SO) contained sulfur-containing compounds such as dimethyl sulfide and thiazole.

### 3.3. Correlation Analysis of Sensory Attributes and Volatile Profiles

**Figure 3:**
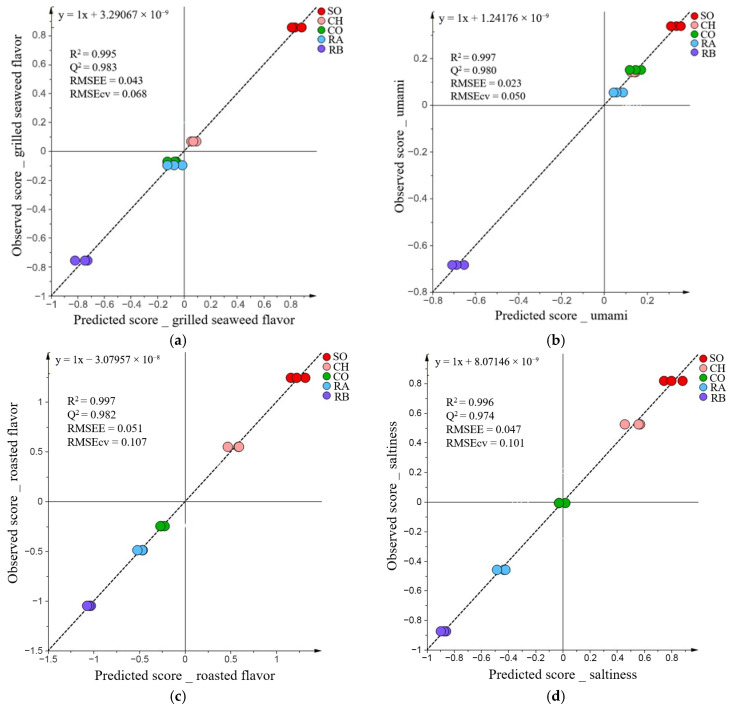
(**a**) OPLSR model performance for grilled seaweed flavor; (**b**) for umami; (**c**) for roasted flavor; (**d**) for saltiness.

To clarify the correlation between sensory attributes and the profiles of volatile compounds in grilled salmonid species, OPLSR models were constructed. The orthogonal components of X variables that do not correlate with the Y variable are separated using OPLSR, which allows easier model interpretation [24]. Therefore, OPLSR analyses were conducted using the four sensory attributes described in Section 3.1. Umami, grilled seaweed flavor, roasted flavor, and saltiness were used as response variables and the volatile profiles of 344 peaks described in Section 3.2 were used as explanatory variables. Figure 3a–d shows the performance of the models. Even for the model with the lowest R^2^ and Q^2^ and the highest RMSEE among the four (0.995, 0.974, and 0.051, respectively), the fit indices indicated good model performance. As noted in Section 3.1, the small sample size (*n* = 5) in this study tends to inflate R^2^ and Q^2^ values. Accordingly, these models were used as exploratory tools to visualize the relationships between sensory attributes and volatile profiles and to screen candidate compounds, rather than as general predictive models.

Subsequently, volatile compounds that correlated with the sensory attributes were extracted based on the VIP and coefficient values. Compounds with VIP > 1 and coefficient > 0 were regarded as useful explanatory variables that were strongly associated with each sensory attribute. This criterion was adopted because our goal was to identify compounds that positively contributed to overall preference. During multivariate analysis, all peaks traceable by MS spectra and retention indices—whether identified, tentatively identified, or unknown—were included as explanatory variables in the OPLSR models. A complete list of peaks meeting VIP > 1 and coefficient > 0 for each sensory attribute is provided in Table S6, with peak numbers and retention indices for unknown compounds shown in Table S5. This analysis identified 87, 97, 82, and 75 compounds associated with grilled seaweed flavor, umami, roasted flavor, and saltiness, respectively. All these compounds were considered robust positive contributors to their respective sensory attributes. They included aldehydes, alcohols, furans, sulfur-containing compounds, hydrocarbons, and other unknown compounds.

### 3.4. Identification of Active Aroma Compounds Using GC/O Analysis

The OPLSR models, which included sensory attributes as response variables and the profiles of 344 peaks as explanatory variables, identified several candidate compounds associated with each attribute. However, the olfactory thresholds of the compounds were not considered in the models. Therefore, whether consumers can perceive these compounds as part of the flavor profile remains unclear. Thus, GC/O analysis was conducted to evaluate the actual olfactory contribution. It should be noted that a high VIP score represents statistical relevance within the model but does not necessarily indicate a direct contribution to aroma perception. Accordingly, only peaks that satisfied VIP > 1 and coefficient > 0 and were perceived in at least one sample during GC/O analysis were designated as key flavor candidates likely to contribute to the characteristic flavor of grilled salmonid species and were subjected to subsequent mechanistic interpretation. Peaks meeting VIP > 1 and coefficient > 0 but not detected by GC/O were considered potentially useful for improving model accuracy; however, their direct contribution to aroma remains unclear and was not discussed mechanistically in this paper. Table 2 shows the key candidate compounds identified by this approach, and Table S5 shows the full GC/O results for all compounds in all samples.

Based on these findings, 23 compounds—including two unknowns—were identified as key contributors. The two unknown peaks were regarded as important candidate compounds in this study and are prioritized as key targets for structural elucidation in future work. Among the remaining 21 compounds, more than half were carbonyl compounds (such as aldehydes and ketones) as well as alcohols. In GC/O analysis, these compounds exhibited aromas described as grilled fish, roasted, or green, and they showed strong positive correlations with “grilled seaweed flavor” and “roasted flavor” in OPLSR analyses. The consistency between the two methods strongly supports the reliability of the results. These 23 compounds were therefore treated as key aroma- active candidates in the subsequent analyses, including odor activity values (OAVs) calculation and additive tests (Section 3.5 and Section 3.6).

### 3.5. Quantification of Key Flavor Candidates

To gain deeper insights into the key flavor candidates responsible for the overall preference of grilled salmonid species, 16 compounds with authentic standards were quantified in SO (sample with the strongest “delicious flavor of grilled salmon”) and CO (intermediate sample) as representative samples. Table 3 shows the results for each compound, and Table S7 shows the parameters of the calibration curves (slope, intercept, and R^2^). All the calibration curves showed good linearity. Dimethyl sulfide, TMA, 1-propanol, cyclohexanone, trans-3-hexen-1-ol, 2-nonanone, 1-heptanol, and 2,6,10,14-tetramethylpentadecane were significantly more abundant in SO than in CO. Notably, the amount of 2,6,10,14-tetramethylpentadecane was approximately 42-fold higher in SO than in CO. Although hydrocarbons generally have high olfactory thresholds, GC/O analysis in this study revealed a roasted-like aroma for 2,6,10,14-tetramethylpentadecane. The OAVs of compounds that were more than 3-fold more abundant in SO than in CO were calculated using published odor thresholds [25]. The resulting OAVs were 14.4, 2848, and 3.1 for dimethyl sulfide, TMA, and 1-heptanol, respectively. Compounds with OAVs greater than 1 were considered to have a strong impact on flavor, and all three exceeded this threshold.

### 3.6. Verification of the Effects of Key Flavor Candidates by Additive Test

To further examine the key flavor candidates most strongly associated with the overall preference for grilled salmonid species, an additive test was conducted based on the quantification results for SO and CO to verify the hypothesis that “the flavor difference between CO and SO is primarily attributable to differences in the concentrations of key flavor candidate compounds.” Four samples—TA, TB, SO, and CO—were ranked according to the intensity of the pleasant grilled salmon aroma and flavor. The average aroma ranks assigned by 16 panelists were as follows: SO: 1.3 ± 0.4, TB: 2.2 ± 0.8, TA: 2.7 ± 0.8, and CO: 3.9 ± 0.3. The average flavor ranks were as follows: SO: 1.3 ± 0.4, TB: 2.1 ± 0.8, TA: 2.8 ± 0.7, and CO: 3.9 ± 0.3. The test samples with added key flavor candidates ranked higher than CO, and TB ranked higher than TA in both aroma and flavor evaluations.

Friedman tests revealed a significant difference among the samples (*p* < 0.05). Pairwise comparisons were performed by calculating ΔRS and comparing them to the LSD, as shown in Table 4. Significant differences were found between SO and CO, and between CO and TB, whereas no significant difference was observed between SO and TB. This indicates that the aroma and flavor of TB are statistically similar to those of SO.

By contrast, TA and SO differed significantly, whereas TA and CO did not. This means that under the conditions of this study, adding only the amount equivalent to the concentration difference between SO and CO was insufficient to statistically improve CO’s flavor to the same level as SO. Although the average rank and ΔRS for CO–TA shifted toward SO, the difference did not meet the significance threshold.

## 4. Discussion

In this study, palatable compounds characterizing the flavor of grilled salmonid species were explored by analyzing correlations between sensory evaluation and volatile compound profiles in five types of grilled salmonid species (SO, CH, CO, RA, and RB) commonly consumed in Japan.

Based on sensory evaluations, PCA revealed that these species could be broadly classified into three groups. The SO and CH group scored high for “delicious flavor of grilled salmon” and positively correlated with the attributes of grilled seaweed flavor, umami, roasted flavor, and saltiness. A total of 344 peaks, including aldehydes, alcohols, and sulfur-containing compounds were detected in the volatile profiles. Their PCA results were consistent with those of the sensory evaluation. When examining the loading plot, terpenes and alcohols were located in the positive direction of PC1 (CO and RA), while sulfur -containing compounds were positioned in the positive direction of PC2 (SO). Terpenes have been detected in farmed rainbow trout and pangasius [26,27], and Podduturi et al. reported that their origin is the accumulation of terpenes from feed. Because CO and RA used in this study were also farmed fish, a similar mechanism may have contributed to their classification. Alcohols are known to be generated through lipid oxidation, and 1-pentanol and 1-octen-3-ol are primarily derived from linoleic acid oxidation [28]. CO and RA exhibited the highest lipid contents among the five species analyzed (Table S8), suggesting that these volatile alcohols contributed to separation along PC1. Furthermore, sulfur-containing compounds such as dimethyl sulfide are known to be produced by thermal degradation of methionine and cysteine [7], indicating that differences in amino acid composition among species may influence the formation of sulfur-containing compounds during heat processing. In the OPLSR using sensory evaluation data and volatile profiles, aldehydes, alcohols, furans, and sulfur-containing compounds were identified as candidate compounds associated with “grilled seaweed flavor”, “umami”, “roasted flavor”, and “saltiness”. These classes of compounds are known to be generated through lipid oxidation and Maillard reactions [29,30]. These findings suggest that the aroma characteristic of grilled salmonid species is primarily derived from precursors such as polyunsaturated fatty acids (PUFAs) and amino acids, and that the resulting volatile compounds may contribute not only to aroma perception but also to taste impressions such as umami and saltiness. Upon incorporating the results of GC/O analysis, 23 compounds—primarily consisting of carbonyl compounds and alcohols—were identified as key flavor candidates. Many of these compounds are thought to be derived primarily from lipids. For example, 2,4-heptadienal is thought to be generated from the thermal degradation or oxidation of PUFAs such as eicosapentaenoic, docosahexaenoic, and α-linolenic acids; propanal from α-linolenic acid; and 1-heptanol and 2-nonanone from linoleic acid or monounsaturated fatty acids (MUFAs) such as oleic acid [29,30,31,32,33,34]. As salmonid species are rich in PUFAs [11], the formation of these carbonyl and related compounds is likely attributable to heating the samples at 250 °C. Generally, odor-active compounds derived from PUFAs are responsible for the off-flavor of fish oil. However, our results suggest that these compounds not only contribute to unpleasant odors but also play roles in characterizing the aroma typical of grilled salmonid species. Among the 23 compounds identified in this study, in addition to those derived from lipids, there were also compounds such as acetaldehyde, dimethyl sulfide, S-methyl thioacetate, and 2-methylpropanal, which may be produced through the decomposition or Strecker degradation of amino acids such as alanine, sulfur-containing amino acids, and valine [30,35]. In particular, dimethyl sulfide and S-methyl thioacetate are considered to substantially contribute to aroma because of their low odor thresholds. Dimethyl sulfide has been detected in marine products such as white-fleshed fish, shrimp, and sea cucumbers and has a characteristic aroma [36,37,38]. By contrast, S-methyl thioacetate has been frequently found in fermented foods such as cheese and beer, whereas occasionally in marine products. The aromatic quality of S-methyl thioacetate in the gonads of sea urchins has been described as “fishy” or “fish oil-like,” suggesting its importance in the characteristic aroma of marine products [39]. As the main precursors of these compounds are sulfur-containing amino acids, they are thought to play significant roles in the formation of the grilled salmonid aroma. Furthermore, 2,6,10,14-tetramethylpentadecane, identified as strongly associated with roasted flavor through GC/O analysis and OPLSR, may originate from copepods consumed by salmonid species as part of their diet. Phytol obtained from phytoplankton by copepods is converted into 2,6,10,14-tetramethylpentadecane via phytanic acid [40,41]. In this study, no data were obtained regarding the diet of the salmon; therefore, the origin of this compound cannot be determined with certainty. However, as one possible hypothesis, phytol may have accumulated in salmon through the food chain and been detected as an aroma compound during grilling. These considerations suggest that, at least for some aroma-active compounds, not only processing conditions such as oven heating but also the dietary history of salmon may influence aroma formation. Modifying the lipid and amino acid composition of feed in aquaculture has been shown to alter the fatty acid and free amino acid composition of fish muscles [42,43,44]. Because fatty and amino acids are precursors of aromatic compounds, manipulating feed composition may enable control over flavorful compounds that characterize marine products. However, this remains a hypothesis derived from the present findings, and systematic evaluation of the relationship between feed composition and muscle 2,6,10,14-tetramethylpentadecane content represents an important topic for future investigation.

The present findings also suggest that perceptions of saltiness and umami may be modulated by aroma compounds. For example, 2-methoxy-4-vinylphenol, which has a smoky aroma, methional, which is associated with a sulfur-like aroma, and pyrazines, which are associated with roasted notes, can enhance saltiness [45]. Compounds such as (Z)-4-heptenal, which has a fish oil-like aroma, and 2-methylbutanal, which has a sugar-like aroma, can enhance umami [46]. In this study, 2-ethyl-5-methylfuran, acetaldehyde, and several other compounds were identified as potential contributors to saltiness and umami perception. The mechanisms by which aroma influences taste perception have been re-ported to involve cross-modal interactions between the gustatory and olfactory systems, as well as direct actions of certain volatile compounds on taste receptors [47,48]. Previous studies have shown that cross-modal enhancement of taste is more likely when the sensory characteristics of odor and taste are congruent [49]. In this study, GC/O analysis indicated that dimethyl sulfide was perceived as a “seaweed-like” odor, which is commonly associated with seawater or salted marine products. Such sensory congruence may have contributed to the enhancement of saltiness. However, based on the present data alone, it is not possible to determine which pathway underlies the observed changes in saltiness and umami. Future work should include additive tests and molecular-level analyses to clarify both the magnitude of this effect and its underlying mechanisms.

Among the 23 identified compounds, TMA, dimethyl sulfide, and 1-heptanol exhibited high OAVs, suggesting that they likely make particularly significant contributions to delicious flavor of grilled salmon. Dimethyl sulfide and TMA have been reported to contribute to seaweed-like and fish-like aromas, respectively [50,51]. Dimethyl sulfide is known to be generated through thermal degradation of sulfur-containing amino acids such as methionine and cysteine, whereas TMA is mainly produced by bacterial reduction and decomposition of trimethylamine oxide (TMAO) [7,51]. In addition, 1-heptanol, which was perceived as a grilled fish-like aroma in GC/O analysis in this study, has been reported to be formed through the degradation of PUFAs and MUFAs such as linoleic and oleic acids [31,32]. Because SO exhibited markedly higher OAVs for these compounds than CO, the distinctive flavor profile observed in SO, including grilled seaweed flavor, is likely explained, at least in part, by sulfur-containing compounds derived from sulfur-containing amino acids, volatile nitrogenous compounds derived from TMAO, and lipid oxidation products derived from PUFAs. It should be noted that seven of the 23 key flavor candidates identified in this study were not quantified because authentic standards were unavailable; however, this does not imply that their contribution to flavor is negligible. These unquantified candidates showed relatively high odor intensities in GC/O analysis and large VIP and coefficient values, suggesting that they may still make an important contribution to the flavor of grilled salmonid species. At present, their impact can only be inferred qualitatively from odor descriptions and relative peak areas. Future work should therefore focus on the structural elucidation of these compounds and the synthesis of authentic standards, which would enable their absolute quantification and OAV estimation.

When some of the identified compounds were added to grilled salmonid species, both TB and TA showed improved rankings in terms of the intensity of pleasant grilled salmon aroma and flavor. In Friedman tests, the aroma and flavor of TB are statistically similar to those of SO. In other words, adding key flavor candidates to CO at high concentrations can partially reproduce the flavor profile characterized by grilled seaweed flavor, roasted flavor, umami, and saltiness, as shown in Section 3.1, even in an actual food system. However, TB contained four times the concentration difference between SO and CO. Therefore, these results provide functional evidence that CO’s flavor profile can be shifted toward SO when key flavor candidates are present at sufficiently high concentrations, but they do not directly prove that the natural flavor difference between SO and CO can be explained solely by the concentration differences in these nine compounds. Although OPLSR modeling and GC/O analysis identified 23 compounds as key contributors to the characteristic flavor of grilled salmonid species, the additive test focused on nine compounds that met practical criteria: permitted for food use in Japan, available in food-grade form, and present at higher concentrations in SO than in CO. Therefore, this test represents a “partial aroma reconstruction” using a representative subset of compounds. The lack of statistical improvement in TA suggests that adding only these nine compounds at the SO–CO difference level cannot fully explain the flavor difference, likely because the contribution of key flavor candidates not included in this reconstruction was not reflected. Furthermore, the significant improvement achieved in TB, despite using the same compounds as TA, suggests that increasing the concentrations of these key flavor candidates can partially compensate for the absence of other compounds and enhance aroma intensity to a level closer to SO. Future work should aim for a more comprehensive aroma reconstruction by including additional compounds or suitable substitutes with similar odor qualities, which may further improve flavor reproduction.

## 5. Conclusions

In this study, we investigated key flavor compounds contributing to the delicious flavor of grilled salmon by integrating volatile profiling and sensory evaluation. From 344 detected volatile compounds, OPLSR combined with GC/O analysis identified 23 key candidates—including TMA, sulfur-containing compounds, and alcohols—that characterize the distinctive flavor of grilled salmonid species. Furthermore, an additive test in which selected compounds were incorporated into grilled salmonid samples at elevated concentrations partially reproduced the characteristic grilled salmon flavor, suggesting that these compounds play a significant role in flavor formation.

The findings of this study provide valuable insights for flavor design and quality improvement of processed products derived from salmonid species, as well as for guiding raw material selection. In addition, the identified compounds may serve as effective markers for evaluating aroma deterioration in salmonid-based products, offering potential applications in optimizing storage and distribution conditions.

These implications should, however, be viewed in light of several limitations. The sensory evaluation and volatile profiling were conducted on only five salmonid species, which represent the Japanese market. The OPLSR models should therefore be regarded as exploratory. Likewise, the volatile compounds identified here should be interpreted as candidate contributors to the delicious flavor of grilled salmon rather than definitive markers applicable to all salmonid products. Also, in this study, we employed trained panels in order to ensure consistency in the definitions of sensory attributes and to obtain stable evaluation scores suitable for multivariate modeling. However, such trained panels do not necessarily represent the general consumer population. Therefore, the sensory evaluation results obtained in this study should be interpreted as indicators of palatability based on expert assessors rather than as a direct reflection of consumer liking. Future studies should include a broader range of salmonid species and production systems (e.g., different geographic origins and both wild and farmed fish) and should use consumer panels with more diverse demographic and cultural backgrounds to verify the external validity of these findings.

## Figures and Tables

**Table 1:** VIP values and coefficients for 13 sensory attributes in the OPLSR model constructed with “delicious flavor of grilled salmon” as the response variable.

Attribute	VIP	Coefficient
grilled seaweed flavor	1.26	0.1693
umami	1.21	0.1335
muddy flavor	1.21	−0.1300
steamy flavor	1.21	−0.1317
burnt flavor	1.20	0.1184
fishy	1.20	−0.1395
saltiness	1.19	0.0967
bloody flavor	1.09	0.0654
green flavor	0.90	−0.0618
sourness	0.69	−0.0123
bitterness	0.48	−0.1091
oily	0.14	0.0559
sweetness	0.04	0.0744

**Table 2:** Key flavor candidate compounds with VIP > 1 and coefficient > 0 in the OPLSR models and detected by GC/O analysis.

No.	Compounds	Identification Method *	Odor Description by GC/O	VIP by OPLSR **			
				Grilled Seaweed	Umami	Roasted	Saltiness
7	Acetaldehyde	MS, RI, STD	Fruity, Sweet	1.73	2.05	1.50	1.65
11	Dimethyl sulfide	MS, RI, STD	Pungent, Seaweed	1.80	1.08	1.76	1.62
15	TMA	MS, RI, STD	Tuna			1.37	1.42
17	Propanal	MS, RI, STD	Green, Fruity	1.31	1.46		
21	2-Methylpropanal	MS, RI, STD	Roasted		1.17	1.35	1.60
57	Unknown	-	Roasted	1.81	1.37	1.47	1.33
72	3-Pentanone	MS, RI, STD	Yogurt				1.04
107	2-Ethyl-5-methylfuran	MS	Roasted	2.02	1.81	1.88	1.89
109	1-Propanol	MS, RI, STD	Vinyl	1.26	1.39		
117	S-Methyl Thioacetate	MS, RI, STD	Fermentation	1.07		1.62	1.74
122	2,3-Pentanedione	MS, RI, STD	Yogurt	1.79	1.80	1.44	1.47
163	2-Ethyl-2-butenal	MS, RI	Paint		1.14		
217	Cyclohexanone	MS, RI, STD	Vinyl				1.09
218	*cis*-2-(2-Pentenyl) furan	MS	Mushrooms	1.14	1.60		
222	*trans*-2-Penten-1-ol	MS, RI	Green	1.25	1.24		
238	*trans*-3-Hexen-1-ol	MS, RI, STD	Paint	1.48	1.43	1.04	
243	*cis*-3-Hexen-1-ol	MS, RI, STD	Green, Grilled fish	1.33	1.25		
248	2-Nonanone	MS, RI, STD	Green, Roasted			1.40	1.52
267	1-Heptanol	MS, RI, STD	Green, Grilled fish			1.08	
269	*trans*, *cis*-2,4-Heptadienal	MS, RI	Grilled fish		1.12		
277	*trans*, *trans*-2,4-Heptadienal	MS, RI, STD	Vinyl		1.13		
300	2,6,10,14-Tetramethylpentadecane	MS, RI, STD	Roasted			1.39	1.54
315	Unknown	-	Cotton candy			1.42	1.56

* Identification method: MS, mass spectrum comparison using NIST 11; STD, confirmed by authentic standards. ** VIP of compounds found to be associated with each sensory attribute.

**Table 3:** Concentrations in SO and CO (μg/kg, average ± SD).

No.	Compounds Name	Concentration (μg/kg)	
		SO	CO
7	Acetaldehyde	7900.42 ± 545.09 ^b^	9626.06 ± 563.98 ^a^
11	Dimethyl sulfide	32.25 ± 6.42 ^a^	1.95 ± 0.01 ^b^
15	TMA	6835.34 ± 192.34 ^a^	1155.23 ± 294.80 ^b^
17	Propanal	20,433.20 ± 2682.42	18,367.55 ± 2413.40
21	2-Methyl propanal	29.41 ± 2.27 ^b^	35.29 ± 0.83 ^a^
72	3-Pentanone	33.47 ± 4.05 ^b^	83.86 ± 6.21 ^a^
109	1-Propanol	424.23 ± 41.09 ^a^	314.49 ± 31.62 ^b^
117	S-Methyl thioacetate	0.65 ± 0.13	0.61 ± 0.08
122	2,3-Pentanedione	5437.58 ± 75.25	5622.35 ± 581.15
217	Cyclohexanone	2.87 ± 0.26 ^a^	1.61 ± 0.05 ^b^
238	*trans* -3-Hexen-1-ol	11.55 ± 1.57 ^a^	7.90 ± 0.42 ^b^
243	*cis*-3-Hexen-1-ol	13.49 ± 0.21	14.04 ± 1.51
248	2-Nonanone	28.84 ± 6.97 ^a^	2.32 ± 0.27 ^b^
267	1-Heptanol	78.29 ± 14.90 ^a^	22.52 ± 2.58 ^b^
277	*trans*, *trans*-2,4-Heptadienal	2873.71 ± 975.98	2284.54 ± 11.92
300	2,6,10,14-Tetramethylpentadecane	92,840.44 ± 2619.88 ^a^	2206.21 ± 613.18 ^b^

Data are mean ± SD across technical replicates (*n* = 3). Different lowercase letters within the same row indicate significant differences at *p* < 0.05, as determined by Welch’s *t*-test. TMA was quantified using the internal standard method, whereas the other compounds were quantified using the standard addition method.

**Table 4:** ΔRS of all sample pairs.

Pairwise Comparison	Aroma ΔRS	Flavor ΔRS
SO—CO	42 *	42 *
SO—TA	23 *	25 *
SO—TB	15	13
CO—TA	19	17
CO—TB	27 *	29 *
TA—TB	8	12

LSD: 19.74, *: Significant (*p* < 0.05).

## Data Availability

Data available in a publicly accessible repository. The raw GC-MS data from this study have been deposited in MB-POST. (https://repository.massbank.jp/preview/11481429826939f129e91f2, accessed on 11 December 2025, PIN CODE: 6815).

## Supplementary Materials

The following supporting information can be downloaded at https://www.mdpi.com/article/10.3390/metabo16010030/s1, Figure S1. Relationship between observed and OPLSR-predicted scale values for the “delicious flavor of grilled salmon” based on sensory evaluation data: Table S1. Sample information for the five salmonid species used in this study: Table S2. Definitions of sensory attributes used for the evaluation of grilled salmonid species: Table S3. Selected ions and concentration ranges of calibration curves used for quantifying key flavor candidates: Table S4. Scale values of sensory evaluation for 14 attributes of grilled salmonid species: Table S5. Volatile compounds detected by non-targeted GC/MS: Table S6. Flavor compounds with VIP > 1 and coefficient > 0 in the sensory attributes about grilled seaweed flavor (a), umami (b), roasted flavor (c), and saltiness (d): Table S7. Calibration curves and R^2^ values for the quantification of flavor candidates in SO and CO: Table S8. Lipid contents in each sample.

## Abbreviations

The following abbreviations are used in this manuscript:

GC/MS	Gas chromatography/mass spectrometry
OPLSR	Orthogonal partial least squares regression
GC/O	Gas chromatography/olfactometry
PCA	Principal component analysis
FAO	Food and Agriculture Organization of the United Nations
MAFF	Ministry of Agriculture, Forestry, and Fisheries
SO	Sockeye salmon
CH	Chum salmon
CO	Coho salmon
RA	Rainbow trout cultured in salt water
RB	Rainbow trout cultured in freshwater
TMA	Trimethylamine
SPME	Solid-phase microextraction
QC	quality control
RI	Retention index
VIP	Variable importance in the projection
RMSEE	Root mean square error of estimation
SIM	Selected ion monitoring
TA	Test A
TB	Test B
ΔRS	Rank sum differences
LSD	The least significant difference
OAVs	Odor activity values
PUFA	Polyunsaturated fatty acids
MUFA	Monounsaturated fatty acids
TMAO	Trimethylamine oxide
