# Supplementary material for: Exploration of Key Flavor Compounds in Five Grilled Salmonid Species by Integrating Volatile Profiling and Sensory Evaluation
Source: Metabolites. 2025 Dec 26;16(1):30. doi: 10.3390/metabo16010030 (PMC12844265; doi:10.3390/metabo16010030)
Supplement: Supplementary file 1 [file metabolites-16-00030-s001.zip › metabolites-4066768-supplementary.pdf]

Supplementary materials

List of contents

Figure S1. Relationship between observed and OPLSR-predicted scale values for the “delicious flavor of grilled salmon” based on sensory evaluation data.

Table S1. Sample information for the five salmonid species used in this study.

Table S2. Definitions of sensory attributes used for the evaluation of grilled salmonid species.

Table S3. Selected ions and concentration ranges of calibration curves used for quantifying key flavor candidates.

Table S4. Scale values of sensory evaluation for 14 attributes of grilled salmonid species.

Table S5. Volatile compounds detected by non-targeted GC/MS.

Table S6. Flavor compounds with VIP＞1 and coefficient＞0 in the sensory attributes about grilled seaweed flavor (a), umami (b), roasted flavor (c), and saltiness (d).

Table S7. Calibration curves and R^2 values for the quantification of flavor candidates in SO and CO.

Table S8. Lipid contents in each sample.

Figure S1. Relationship between observed and OPLSR-predicted scale values for the "delicious flavor of grilled salmon" based on sensory evaluation data.

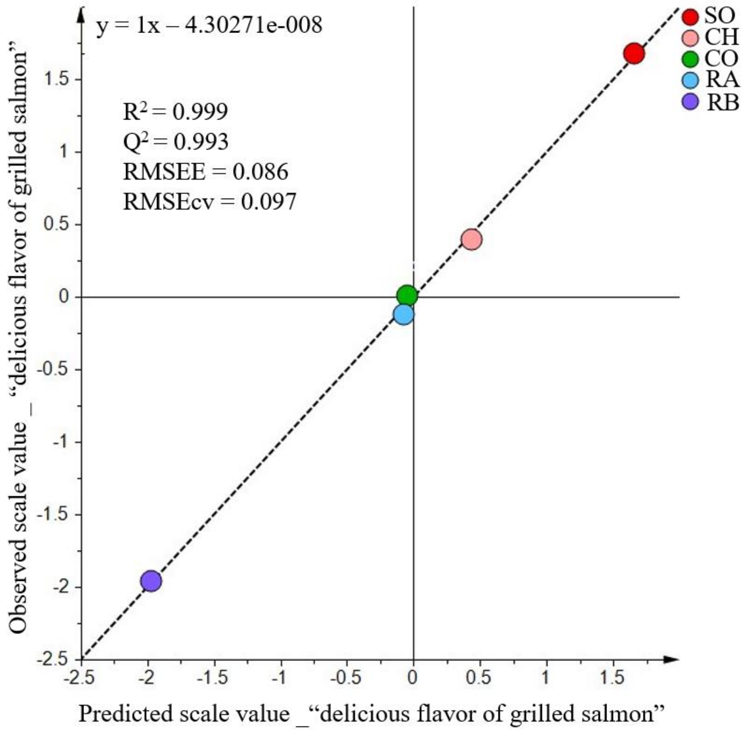

Table S1. Sample information for the five salmonid species used in this study.

Sample name	Species	Japanese name	Wild / Cultured	Fishing season	Size
SO	Sockeye salmon	Benizake	Wild	Unidentified	2.0 – 2.1 kg*
CH	Chum salmon	Shirozake	Wild	Fall, 2022	1.5 – 1.8 kg**
CO	Coho salmon	Ginzake	Cultured	Fall, 2022	2.1 – 2.7 kg**
RA	Rainbow trout	Salmon-trout	Cultured	Fall, 2022	2.5 – 2.7 kg**
RB		Nijimasu	Cultured	Unidentified	0.09 – 0.1 kg*

Note: *Weight without internal organs

**Weight without head and internal organs

Table S2. Definitions of sensory attributes used for the evaluation of grilled salmonid species.

Attribute	Definition
sweetness	The five basic tastes
umami	The five basic tastes
saltiness	The five basic tastes
sourness	The five basic tastes
bitterness	The five basic tastes
oily	Sticky and oily feel
roasted flavor	Roasted, grilled
fishy	Ammonia-like, raw fish flavor
grilled seaweed flavor	Grilled seaweed-like
bloody flavor	Iron-like
muddy flavor	Clay-like
green flavor	Cucumber-like
steamy flavor	Flavor that lingers in the nose like white fish
delicious flavor of grilled salmon	The appetizing flavor of grilled salmon

Table S3. Selected ions and concentration ranges of calibration curves used for quantifying key flavor candidates.

RI	Compounds name	m/z	Concentration ranges ppm (w/v)
78	Acetaldehyde	44	0 – 10
740	Dimethyl sulfide	62	0 – 0.25
806	Propanal	58	0 – 50
822	2-Methyl propanal	72	0 – 0.25
992	3-Pentanone	57	0 – 0.5
1045	1-Propanol	31	0 – 0.5
1064	S-Methyl thioacetate	90	0 – 0.01
1073	2,3-Pentanedione	43	0 – 10
1305	Cyclohexanone	98	0 – 0.005
1369	*trans*-3-Hexen-1-ol	67	0 – 0.1
1390	*cis*-3-Hexen-1-ol	67	0 – 0.1
1397	2-Nonanone	58	0 – 0.5
1457	1-Heptanol	70	0 – 0.5
1507	*trans, trans*-2,4-Heptadienal	81	0 – 10
1656	2,6,10,14-Tetramethylpentadecane	57	0 – 200

Table S4. Scale values of sensory evaluation for 14 attributes of grilled salmonid species.

Attribute	SO	CH	CO	RA	RB
sweetness	-0.14	-0.26	0.27	0.35	-0.22
umami	0.34	0.14	0.15	0.05	-0.68
saltiness	0.82	0.52	-0.01	-0.46	-0.87
sourness	-0.10	0.36	0.30	-0.05	-0.51
bitterness	0.02	0.09	-0.07	-0.29	0.25
oily	-0.62	-0.97	0.93	1.17	-0.51
roasted flavor	1.24	0.55	-0.25	-0.49	-1.05
fishy	-0.84	-0.24	0.27	0.19	0.61
grilled seaweed flavor	0.86	0.07	-0.07	-0.10	-0.76
bloody flavor	0.20	0.30	0.20	-0.13	-0.57
muddy flavor	-0.87	-0.30	0.19	0.29	0.68
green flavor	-0.53	-0.38	0.37	0.30	0.24
steamy flavor	-0.99	-0.34	0.27	0.29	0.77
delicious flavor of grilled salmon	1.68	0.39	0.01	-0.12	-1.96

Table S5. Volatile compounds detected by non-targeted GC/MS.

No.	RI [a]	Compounds	Identification method [b]	Odor description	Odor intensity [c]				
					SO	CH	CO	RA	RB
1	555	Unknown	-						
2	629	Hexane, 2-methyl-	MS						
3	640	Unknown	-						
4	653	Methanethiol	MS, RI	French fries	3.3	3.0	2.5	2.8	4.0
5	661	Heptane	MS, RI, STD						
6	664	Unknown	-						
7	678	Acetaldehyde	MS, RI, STD	Fruity, Sweet		2.0	2.5	2.3	2.0
8	712	Unknown	-						
9	715	1-Heptene	MS, RI						
10	729	Unknown	-						
11	740	Dimethyl sulfide	MS, RI, STD	Pungent		1.0		1.0	1.0
12	750	3-Methylheptane	MS						
13	757	(E)-2-Methyl-1,3-pentadiene	MS						
14	763	Unknown	-						
15	775	TMA	MS, RI, STD	Tuna	1.5	3.0	2.0	1.8	3.8
16	801	Octane	MS, RI, STD						
17	806	Propanal	MS, RI, STD	Green, Fruity	1.7	1.0	1.0	1.8	1.8
18	806	Unknown	-						
19	817	*cis*-1,3-Dimethylcyclohexane	MS						
20	817	Unknown	-						
21	822	2-Methylpropanal	MS, RI, STD	Roasted	1.0			1.5	
22	827	Acetone	MS, RI						
23	840	(Z)-2-Octene	MS, RI						
24	852	4-methyloctane	MS, RI						
25	852	Unknown	-						
26	858	Unknown	-						
27	859	Unknown	-						
28	862	Unknown	-						
29	867	Unknown	-						
30	868	Unknown	-						
31	871	Unknown	-						
32	883	2-Methylfuran	MS, RI, STD						
33	887	Cyclohexane, 1,3,5-trimethyl-	MS						
34	890	Butyraldehyde	MS, RI, STD						
35	892	Unknown	-						

36	900	Unknown	-						
37	900	Nonane	MS, RI, STD	Roasted		0.5			
38	901	Ethyl acetate	MS, RI, STD						
39	909	Unknown	-						
40	909	Unknown	-						
41	914	2-Butanone	MS, RI, STD						
42	914	Unknown	-						
43	917	Unknown	-						
44	918	Unknown	-						
45	919	Unknown	-						
46	920	Unknown	-						
47	924	Unknown	-						
48	924	2-Methylbutanal	MS, RI, STD						
49	924	2,4-Octadiene	MS, RI						
50	925	Unknown	-						
51	926	Unknown	-						
52	928	3-Methylbutanal	MS, RI, STD	Fermentation		1.0			2.5
53	929	*cis*-1-Ethyl-3-methyl-cyclohexane	MS						
54	935	Unknown	-						
55	935	(Z, Z)- 3,5-Octadiene	MS						
56	937	Unknown	-						
57	939	Unknown	-	Roasted				1.0	2.0
58	942	Ethanol	MS, RI, STD						
59	947	Unknown	-						
60	948	Unknown	-						
61	948	Heptane, 2,2,4,6,6-pentamethyl-	MS						
62	953	Benzene	MS, RI						
63	957	Unknown	-						
64	964	2-Ethylfuran	MS, RI, STD						
65	973	Unknown	-						
66	968	Unknown	-						
67	972	Unknown	-						
68	980	Unknown	-						1.0
69	981	Unknown	-						
70	981	Propylcyclohexane	MS						
71	985	Unknown	-						
72	991	3-Pentanone	MS, RI, STD	Yogurt	1.0	1.5	1.0	2.0	1.0
73	993	Pentanal	MS, RI, STD						
74	994	Unknown	-						

75	998	Unknown	-						
76	999	Decane	MS, RI, STD						
77	1000	Unknown	-						
78	1001	Unknown	-						
79	1006	Unknown	-						
80	1007	Unknown	-						
81	1008	Unknown	-						
82	1008	Unknown	-						
83	1010	Unknown	-						
84	1010	Unknown	-						
85	1010	Unknown	-						
86	1011	Unknown	-						
87	1013	Unknown	-						
88	1015	*trans*1-Methyl-4-(1-methylethyl)-cyclohexane	MS						
89	1016	Unknown	-						
90	1018	Unknown	-	Potato	1.0	1.0			
91	1020	Unknown	-						
92	1020	1-Methyl-3-propyl-cyclohexane	MS	Yogurt					1.0
93	1023	4-Methyl-1,3-heptadiene	MS						
94	1025	α-Pinene	MS, RI, STD						
95	1026	Unknown	-						
96	1028	Unknown	-						
97	1028	Unknown	-						
98	1031	Unknown	-						
99	1031	Unknown	-						
100	1032	Chloroform	MS, RI						
101	1034	1-Penten-3-one	MS, RI						
102	1037	Unknown	-						
103	1037	Unknown	-						
104	1039	Unknown	-	Roasted					1.0
105	1039	Unknown	-						
106	1039	Unknown	-						
107	1042	2-Ethyl-5-methylfuran	MS	Roasted		1.0		1.0	
108	1042	Unknown	-						
109	1046	1-Propanol	MS, RI, STD	Vinyl		0.8		0.5	
110	1046	Unknown	-						
111	1052	Toluene	MS, RI						
112	1051	Unknown	-						
113	1053	Unknown	-						

114	1057	2-Butenal	MS, RI, STD						
115	1059	(E, E)- 1,3,6-Octatriene	MS, RI	Roasted		0.8			
116	1059	Unknown	-						
117	1062	S-Methyl Thioacetate	MS, RI, STD	Fermentation					1.0
118	1064	Unknown	-						
119	1064	Unknown	-						
120	1066	Unknown	-						
121	1069	Camphene	MS, RI, STD						
122	1072	2,3-Pentanedione	MS, RI, STD	Yogurt	3.0			2.2	2.0
123	1073	Unknown	-						
124	1073	Unknown	-						
125	1076	Butylcyclohexane	MS, RI						
126	1082	Unknown	-						
127	1085	Unknown	-						
128	1088	Undecane	MS, RI, STD						
129	1089	Unknown	-						
130	1093	Hexanal	MS, RI, STD	Green, Apple	2.0	1.3	3.5	3.3	2.7
131	1097	Unknown	-						
132	1104	Unknown	-						
133	1107	β-Pinene	MS, RI						
134	1108	Unknown	-						
135	1108	(E, Z)-4-Ethylidenecyclohexene	MS						
136	1110	2-Methyl-2-butenal	MS						
137	1110	Unknown	-						
138	1112	Unknown	-						
139	1117	3-Pentanol	MS, RI, STD						
140	1122	Unknown	-	Pungent			1.5	1.5	
141	1122	Unknown	-						
142	1122	Unknown	-						
143	1126	Unknown	-						
144	1128	Unknown	-						
145	1129	Unknown	-						
146	1130	Unknown	-						
147	1131	Unknown	-						
148	1135	Ethyl benzene	MS, RI						
149	1136	Unknown	-						
150	1137	Unknown	-						
151	1140	Unknown	-						
152	1141	2,3-Hexanedione	MS, RI						

153	1142	Unknown	-	Sweet and Roasted				1.5
154	1144	*p*-Xylene	MS, RI					
155	1144	*trans*-2-Pentenal	MS, RI, STD					
156	1144	Unknown	-					
157	1149	Unknown	-					
158	1150	1,3-Dimethylbenzene	MS, RI	Vinyl		2.0	2.0	
159	1152	Unknown	-					
160	1152	Unknown	-					
161	1154	Decahydromaphthalene	MS					
162	1155	Unknown	-					
163	1162	2-Ethyl-2-butenal	MS, RI	Paint	1.0			
164	1166	Unknown	-					
165	1167	Unknown	-					
166	1167	1-Penten-3-ol	MS, RI, STD					
167	1177	Unknown	-					
168	1181	Unknown	-					
169	1182	α-Terpinene	MS, RI					
170	1184	Thiophene, 2-ethyl-	MS, RI, STD					
171	1190	Unknown	-					
172	1190	Dodecane	MS, RI, STD					
173	1192	2-Heptanone	MS, RI, STD					
174	1195	Heptanal	MS, RI, STD					
175	1195	Unknown	-					
176	1197	Unknown	-					
177	1198	Unknown	-					
178	1202	Unknown	-					
179	1203	D-Limonene	MS, RI, STD					
180	1205	Unknown	-					
181	1212	β-Phellandrene	MS, RI					
182	1215	Unknown	-					
183	1217	Unknown	-					
184	1217	Propylbenzene	MS, RI					
185	1224	Unknown	-					
186	1230	Unknown	-	Roasted			1.0	
187	1231	Unknown	-	Floral	0.5			
188	1231	*trans*-2-Hexenal	MS, RI, STD					
189	1234	Unknown	-					
190	1233	Unknown	-					
191	1236	Unknown	-					

192	1238	2-Pentylfuran	MS, RI					
193	1244	Unknown	-					
194	1247	4-Heptenal	MS, RI, STD					
195	1251	γ-Terpinene	MS, RI, STD					
196	1252	Unknown	-					
197	1253	Unknown	-	Muddy	1.0		1.0	
198	1254	1,3,5-Trimethylbenzene	MS, RI	Steamy				1.0
199	1255	1-Pentanol	MS, RI, STD					
200	1256	Unknown	-					
201	1257	Unknown	-					
202	1263	Unknown	-	Fruity			1.0	
203	1264	Thiazole	MS, RI, STD					
204	1264	Unknown	-					
205	1265	Unknown	-					
206	1266	Unknown	-					
207	1267	Unknown	-					
208	1269	Unknown	-					
209	1270	Styrene	MS, RI					
210	1272	1-Ethyl-4-methylbenzene	MS					
211	1278	*p*-Cymene	MS, RI					
212	1289	2-Carene	MS, RI					
213	1289	Unknown	-					
214	1293	1,2,4-Trimethylbenzene	MS, RI					
215	1293	1,2,3-Trimethylbenzene	MS					
216	1298	Octanal	MS, RI					
217	1305	Cyclohexanone	MS, RI, STD	Vinyl			0.5	
218	1311	*cis*-2-(2-Pentenyl) furan	MS				2.0	
219	1312	Unknown	-					
220	1312	Unknown	-					
221	1315	Unknown	-					
222	1319	*trans*-2-Penten-1-ol	MS, RI	Mushrooms		1.5		
223	1322	Butylbenzene	MS, RI					
224	1323	Unknown	-					
225	1327	*cis*-2-Penten-1-ol	MS, RI, STD	Iron		1.0		
226	1334	4-Nonanone	MS	Smoky			1.0	
227	1334	Unknown	-					
228	1337	Unknown	-					
229	1342	Unknown	-	Fruity				1.5
230	1344	Unknown	-					

231	1345	1-Ethyl-2-methylbenzene	MS, RI						
232	1348	Unknown	-						
233	1349	Unknown	-						
234	1355	2-Methyl-3-pentanol,	MS, RI						
235	1355	Unknown	-						
236	1358	1-Hexanol_111-27-3	MS, RI, STD						
237	1364	4-Ethyl-1,2-dimethylbenzene	MS, RI						
238	1369	*trans*-3-Hexen-1-ol	MS, RI, STD	Paint				1.7	
239	1370	Unknown	-						
240	1370	1-Ethyl-2,3-dimethylbenzene	MS, RI						
241	1378	4-Ethyl-1,2-dimethylbenzene	MS, RI	Pungent		0.8			
242	1383	Unknown	-						
243	1390	*cis*-3-Hexen-1-ol	MS, RI, STD	Green, Grilled fish	2.5	3.2	3.3	3.8	3.7
244	1391	Unknown	-						
245	1392	Tetradecane	MS, RI, STD						
246	1392	Unknown	-						
247	1395	Dimethyl Trisulfide	MS, RI						
248	1397	2-Nonanone	MS, RI, STD	Green, Roasted	1.0	1.8	2.0		
249	1402	Nonanal	MS, RI, STD	Paint	2.0		2.7	0.8	
250	1407	Unknown	-						
251	1408	Unknown	-						
252	1415	Trimethylpyrazine	MS, RI, STD						
253	1418	3-Octen-2-one	MS, RI, STD						
254	1421	(E, E)-2,4-Hexadienal	MS, RI						
255	1421	Unknown	-						
256	1426	Unknown	-	Green		1.3	1.0	0.5	
257	1429	3-Ethyl-2-methyl-1,3-hexadiene	MS, RI				1.5		
258	1431	Unknown	-						
259	1441	Unknown	MS, RI, STD						
260	1446	Unknown	-						
261	1447	Unknown	-	Pungent				1.0	
262	1448	1,2,3,4-Tetramethylbenzene	MS, RI						
263	1449	Unknown	-	Soy sauce				1.0	
264	1452	1-Octen-3-ol	MS, RI, STD	Steamy	1.0	1.5	2.3	2.2	
265	1455	Unknown	-						
266	1456	Unknown	-						
267	1458	1-Heptanol	MS, RI, STD	Green, Grilled fish	2.0	2.5	1.7	2.3	1.8
268	1462	Unknown	-						
269	1478	*trans, cis*-2,4-Heptadienal	MS, RI	Grilled fish	1.0	0.8	1.5	1.0	2.0

270	1480	Furfural	MS, RI, STD					2.0	
271	1487	Propanoic acid, 2,2-dimethyl-, 2-ethylhexyl ester	MS						
272	1488	Unknown	-						
273	1488	Pentadecane	MS, RI, STD						
274	1500	Unknown	-						
275	1501	Unknown	-						
276	1504	Decanal	MS, RI, STD						
277	1507	*trans, trans*-2,4-Heptadienal	MS, RI, STD	Vinyl				2.0	
278	1510	Unknown	-						
279	1512	Unknown	-						
280	1520	Unknown	-						
281	1528	Unknown	-						
282	1531	3,5-Octadien-2-one	MS, RI	Pork bone					1.3
283	1535	Unknown	-						
284	1542	Benzaldehyde	MS, RI, STD	Bunt	2.5	2.0	0.8	2.0	
285	1551	Unknown	-						
286	1561	1-Octanol	MS, RI, STD	Steamy			1.0		0.5
287	1568	Unknown	-						
288	1586	(E, E)-3,5-Octadien-2-one	MS, RI	Bunt			0.5		
289	1591	Hexadecane	MS, RI, STD						
290	1600	Unknown	-						
291	1602	Unknown	-						
292	1602	Caralene	MS, RI						
293	1607	2-Undecanone	MS, RI	Fruity					2.0
294	1612	4-Methyl-3-pentenoic acid	MS						
295	1614	Undecanal	MS, RI, STD						
296	1624	Unknown	-						
297	1631	Unknown	-						
298	1655	Cyclohexanol, 1-ethynyl-	MS, RI, STD						
299	1657	Unknown	-						
300	1657	2,6,10,14-Tetramethylpentadecane	MS, RI, STD	Roasted			1.0		1.7
301	1666	Unknown	-						
302	1685	Unknown	-						
303	1694	Heptadecane	MS, RI, STD						
304	1694	Unknown	-						
305	1695	Unknown	-						
306	1695	Unknown	-						
307	1698	Unknown	-						
308	1709	1,2,3,4-Tetrahydro-1,1,6-trimethyl-naphthalene	MS, RI						

309	1724	Unknown	-	Green				0.5	
310	1722	2-Thiophenecarboxaldehyde	MS, RI, STD						
311	1734	4-Ethylbenzaldehyde	MS, RI	Green, Fruity		2.7			3.5
312	1734	α-Muurolene	MS, RI						
313	1748	Unknown	-						
314	1752	Unknown	-						
315	1759	Unknown	-	Cotton candy		1.0	1.5		1.0
316	1767	Unknown	-						
317	1789	(E)-4-Oxohex-2-enal	MS	Roasted	1.8	2.7	2.8	3.0	2.8
318	1794	N, N-dibutylformamide	MS, RI						
319	1797	Octadecane	MS, RI, STD						
320	1804	Unknown	-	Rotten egg		1.0			
321	1860	Hexanoic acid	MS, RI, STD	Steamy		1.3			1.8
322	1872	Unknown	-						
323	1893	Unknown	-						
324	1898	Nonadecane	MS, RI, STD						
325	1922	Unknown	-	Fruity					1.5
326	1931	Butylated hydroxytoluene	MS, RI						
327	1937	Unknown	-	Steamy			1.0	1.0	
328	1970	Unknown	-	Green					1.0
329	2061	Ethyl tetradecanoate	MS, RI, STD	Pungent					
330	2066	Unknown	-						1.0
331	2078	Unknown	-						
332	2097	Unknown	-						
333	2104	Unknown	-						
334	2110	Unknown	-						
335	2170	Unknown	-						
336	2206	Unknown	-						
337	2267	Ethyl hexadecanoate	MS, RI, STD						
338	2586	Unknown	-	Roasted	1.0				
339	2623	Unknown	-						
340	2665	Unknown	-						
341	2691	Unknown	-						
342	2732	Unknown	-						
343	2736	Unknown	-						
344	2738	Unknown	-						

a Retention index: calculated using standard of n-alkane (C7-33)

b Identification method: MS, mass spectrum comparison using NIST 11; STD, confirmed by authentic standards

c Average of triplicate

Table S6. Flavor compounds with VIP＞1 and coefficient＞0 in the sensory attributes about grilled seaweed flavor (a), umami (b), roasted flavor (c), and saltiness (d).

(a) Grilled seaweed flavor

No.	Compound	VIP	Coefficient
132	Unknown	2.07	0.0133
107	2-Ethyl-5-methylfuran	2.02	0.0127
36	Unknown	1.95	0.0119
49	2,4-Octadiene	1.92	0.0116
55	(Z, Z)- 3,5-Octadiene	1.89	0.0117
50	Unknown	1.89	0.0117
64	2-Ethylfuran	1.89	0.0116
87	Unknown	1.88	0.0121
294	4-Methyl-3-pentenoic acid	1.84	0.0118
196	Unknown	1.84	0.0119
38	Ethyl acetate	1.84	0.0116
78	Unknown	1.83	0.0113
207	Unknown	1.83	0.0118
13	(E)-2-Methyl-1,3-Pentadiene	1.82	0.0116
37	Nonane	1.82	0.0115
57	Unknown	1.81	0.0106
11	Dimethyl sulfide	1.80	0.0113
122	2,3-Pentanedione	1.79	0.0109
18	Unknown	1.79	0.0116
93	4-Methyl-1,3-heptadiene	1.79	0.0111
5	Heptane	1.78	0.0117
56	Unknown	1.78	0.0109
59	Unknown	1.76	0.0108
131	Unknown	1.75	0.0110
242	Unknown	1.75	0.0101
125	Butylcyclohexane	1.75	0.0103
251	Unknown	1.74	0.0102
7	Acetaldehyde	1.73	0.0112
168	Unknown	1.72	0.0095
129	Unknown	1.71	0.0112
79	Unknown	1.69	0.0105
115	(E, E)- 1,3,6-Octatriene	1.69	0.0107
138	Unknown	1.69	0.0097
32	2-Methylfuran	1.68	0.0092
116	Unknown	1.65	0.0102
210	1-Ethyl-4-methylbenzene	1.60	0.0082
148	Ethyl benzene	1.60	0.0082
193	Unknown	1.60	0.0083
48	2-Methylbutanal	1.59	0.0108
221	Unknown	1.59	0.0089
184	Propylbenzene	1.58	0.0081
62	Benzene	1.58	0.0103
231	1-Ethyl-2-methylbenzene	1.58	0.0090
128	Undecane	1.57	0.0082
6	Unknown	1.55	0.0084
233	Unknown	1.52	0.0076

175	Unknown	1.52	0.0074
255	Unknown	1.52	0.0079
241	4-Ethyl-1,2-dimethylbenzene	1.51	0.0080
1	Unknown	1.50	0.0090
234	2-Methyl-3-pentanol	1.50	0.0084
230	Unknown	1.50	0.0090
51	Unknown	1.49	0.0098
41	2-Butanone	1.48	0.0103
238	*trans*-3-Hexen-1-ol	1.48	0.0077
28	Unknown	1.47	0.0095
220	Unknown	1.47	0.0081
240	1-Ethyl-2,3-dimethylbenzene	1.47	0.0077
166	1-Penten-3-ol	1.47	0.0074
22	Acetone	1.46	0.0103
223	Butylbenzene	1.45	0.0081
46	Unknown	1.44	0.0105
71	Unknown	1.40	0.0069
237	4-ethyl-1,2-dimethylbenzene	1.40	0.0071
145	Unknown	1.35	0.0066
243	*cis*-3-Hexen-1-ol	1.33	0.0066
17	Propanal	1.31	0.0067
190	Unknown	1.31	0.0060
164	Unknown	1.26	0.0063
109	1-Propanol	1.26	0.0064
287	Unknown	1.25	0.0073
222	*trans*-2-Penten-1-ol	1.25	0.0071
85	Unknown	1.23	0.0082
206	Unknown	1.21	0.0067
189	Unknown	1.20	0.0054
239	Unknown	1.17	0.0059
101	1-Penten-3-one	1.16	0.0065
66	Unknown	1.15	0.0057
102	Unknown	1.15	0.0055
218	*cis*-2-(2-Pentenyl) furan	1.14	0.0060
279	Unknown	1.14	0.0056
227	Unknown	1.14	0.0056
265	Unknown	1.09	0.0059
203	Thiazole	1.08	0.0081
117	S-Methyl Thioacetate	1.07	0.0085
224	Unknown	1.05	0.0046
170	Thiophene, 2-ethyl-	1.01	0.0075

(b) Umami

No.	Compound	VIP	Coefficient
7	Acetaldehyde	2.05	0.0141
1	Unknown	1.88	0.0120
64	2-Ethylfuran	1.88	0.0111
107	2-Ethyl-5-methylfuran	1.81	0.0113
122	2,3-Pentanedione	1.80	0.0104
32	2-Methylfuran	1.69	0.0091

132	Unknown	1.66	0.0108
78	Unknown	1.62	0.0084
218	*cis*-2-(2-Pentenyl) furan	1.60	0.0111
234	2-Methyl-3-pentanol	1.60	0.0085
131	Unknown	1.60	0.0083
13	(E)-2-Methyl-1,3-pentadiene	1.60	0.0108
115	(E, E)- 1,3,6-Octatriene	1.60	0.0084
145	Unknown	1.59	0.0091
193	Unknown	1.55	0.0073
102	Unknown	1.55	0.0087
66	Unknown	1.54	0.0097
17	Propanal	1.46	0.0068
101	1-Penten-3-one	1.44	0.0076
238	*trans*-3-Hexen-1-ol	1.43	0.0067
41	2-Butanone	1.43	0.0128
116	Unknown	1.42	0.0066
223	Butylbenzene	1.41	0.0060
265	Unknown	1.40	0.0066
221	Unknown	1.40	0.0057
184	Propylbenzene	1.39	0.0056
109	1-Propanol	1.39	0.0061
28	Unknown	1.38	0.0068
57	Unknown	1.37	0.0059
220	Unknown	1.37	0.0055
85	Unknown	1.37	0.0075
230	Unknown	1.37	0.0059
22	Acetone	1.36	0.0124
36	Unknown	1.36	0.0076
206	Unknown	1.36	0.0078
189	Unknown	1.33	0.0056
239	Unknown	1.33	0.0059
5	Heptane	1.31	0.0099
18	Unknown	1.31	0.0093
49	2,4-Octadiene	1.31	0.0064
108	Unknown	1.30	0.0121
231	1-Ethyl-2-methylbenzene	1.30	0.0048
241	4-Ethyl-1,2-dimethylbenzene	1.28	0.0045
240	1-Ethyl-2,3-dimethylbenzene	1.27	0.0044
251	Unknown	1.27	0.0049
6	Unknown	1.26	0.0047
38	Ethyl acetate	1.25	0.0080
243	*cis*-3-Hexen-1-ol	1.25	0.0048
207	Unknown	1.25	0.0083
112	Unknown	1.24	0.0071
55	(Z, Z)- 3,5-Octadiene	1.24	0.0064
287	Unknown	1.24	0.0096
190	Unknown	1.24	0.0042
87	Unknown	1.24	0.0068
222	*trans*-2-Penten-1-ol	1.24	0.0050
34	Butyraldehyde	1.23	0.0080
50	Unknown	1.23	0.0065
237	4-ethyl-1,2-dimethylbenzene	1.23	0.0041
148	Ethyl benzene	1.22	0.0048

255	Unknown	1.22	0.0039
227	Unknown	1.22	0.0043
166	1-Penten-3-ol	1.22	0.0039
37	Nonane	1.22	0.0080
310	2-Thiophenecarboxaldehyde	1.21	0.0062
175	Unknown	1.21	0.0042
168	Unknown	1.21	0.0044
210	1-Ethyl-4-methylbenzene	1.21	0.0040
125	Butylcyclohexane	1.20	0.0045
294	4-Methyl-3-pentenoic acid	1.19	0.0073
62	Benzene	1.19	0.0094
170	Thiophene, 2-ethyl-	1.19	0.0121
21	2-Methylpropanal	1.17	0.0128
233	Unknown	1.17	0.0035
196	Unknown	1.15	0.0065
163	2-Ethyl-2-butenal	1.14	0.0054
138	Unknown	1.14	0.0038
279	Unknown	1.13	0.0035
92	1-Methyl-3-propyl-cyclohexane	1.13	0.0058
277	*trans, trans*-2,4-Heptadienal	1.13	0.0053
97	Unknown	1.12	0.0055
224	Unknown	1.12	0.0035
129	Unknown	1.12	0.0065
269	*trans, cis*-2,4-Heptadienal	1.12	0.0055
68	Unknown	1.11	0.0060
54	Unknown	1.11	0.0069
46	Unknown	1.10	0.0103
44	Unknown	1.09	0.0065
93	4-Methyl-1,3-heptadiene	1.08	0.0047
11	Dimethyl sulfide	1.08	0.0052
198	1,3,5-Trimethylbenzene	1.08	0.0032
242	Unknown	1.07	0.0040
56	Unknown	1.06	0.0046
48	2-Methylbutanal	1.05	0.0084
69	Unknown	1.05	0.0055
155	*trans*-2-Pentenal	1.04	0.0042
214	1,2,4-Trimethylbenzene	1.04	0.0029
59	Unknown	1.03	0.0043

(c) Roasted flavor

No.	Compound	VIP	Coefficient
132	Unknown	2.07	0.0143
5	Heptane	2.04	0.0128
18	Unknown	2.01	0.0132
207	Unknown	1.99	0.0137
38	Ethyl acetate	1.99	0.0125
37	Nonane	1.98	0.0125
294	4-Methyl-3-pentenoic acid	1.96	0.0130
48	2-Methylbutanal	1.94	0.0125
36	Unknown	1.92	0.0119
196	Unknown	1.89	0.0131
107	2-Ethyl-5-methylfuran	1.88	0.0125
46	Unknown	1.88	0.0135

87	Unknown	1.87	0.0130
50	Unknown	1.87	0.0119
62	Benzene	1.86	0.0129
55	(Z, Z)- 3,5-Octadiene	1.84	0.0118
22	Acetone	1.84	0.0119
41	2-Butanone	1.84	0.0118
13	(E)-2-Methyl-1,3-pentadiene	1.84	0.0107
51	Unknown	1.82	0.0118
49	2,4-Octadiene	1.78	0.0112
129	Unknown	1.76	0.0118
11	Dimethyl sulfide	1.76	0.0118
56	Unknown	1.67	0.0111
93	4-Methyl-1,3-heptadiene	1.67	0.0114
59	Unknown	1.66	0.0110
117	S-Methyl Thioacetate	1.62	0.0120
79	Unknown	1.62	0.0109
203	Thiazole	1.61	0.0114
242	Unknown	1.56	0.0094
64	2-Ethylfuran	1.56	0.0108
7	Acetaldehyde	1.50	0.0108
57	Unknown	1.47	0.0089
78	Unknown	1.47	0.0111
76	Decane	1.45	0.0101
122	2,3-Pentanedione	1.44	0.0099
125	Butylcyclohexane	1.44	0.0098
315	Unknown	1.42	0.0097
170	Thiophene, 2-ethyl-	1.41	0.0112
248	2-Nonanone	1.40	0.0098
299	Unknown	1.39	0.0101
300	2,6,10,14-Tetramethylpentadecane	1.39	0.0100
287	Unknown	1.39	0.0088
251	Unknown	1.37	0.0092
168	Unknown	1.37	0.0080
15	TMA	1.37	0.0101
131	Unknown	1.37	0.0107
128	Undecane	1.35	0.0050
21	2-Methylpropanal	1.35	0.0098
138	Unknown	1.34	0.0087
42	Unknown	1.33	0.0107
174	Heptanal	1.29	0.0083
32	2-Methylfuran	1.28	0.0073
115	(E, E)- 1,3,6-Octatriene	1.28	0.0106
71	Unknown	1.27	0.0054
148	Ethyl benzene	1.27	0.0066
313	Unknown	1.26	0.0092
116	Unknown	1.24	0.0097
337	Ethyl hexadecanoate	1.23	0.0065
295	Undecanal	1.22	0.0083
194	4-Heptenal	1.18	0.0086
336	Unknown	1.17	0.0084
210	1-Ethyl-4-methylbenzene	1.16	0.0054
1	Unknown	1.13	0.0072
193	Unknown	1.12	0.0057

6	Unknown	1.11	0.0065
184	Propylbenzene	1.10	0.0055
221	Unknown	1.10	0.0073
164	Unknown	1.10	0.0053
324	Nonadecane	1.10	0.0058
231	1-Ethyl-2-methylbenzene	1.10	0.0075
298	Cyclohexanol, 1-ethynyl-	1.09	0.0099
175	Unknown	1.09	0.0043
267	1-Heptanol	1.08	0.0060
233	Unknown	1.06	0.0045
28	Unknown	1.05	0.0099
234	2-Methyl-3-pentanol	1.04	0.0071
238	*trans*-3-Hexen-1-ol	1.04	0.0058
255	Unknown	1.01	0.0056
209	Styrene	1.01	0.0072
230	Unknown	1.01	0.0085
329	Ethyl tetradecanoate	1.00	0.0054

(d) Saltiness

No.	Compound	VIP	Coefficient
132	Unknown	2.05	0.0142
5	Heptane	2.03	0.0128
46	Unknown	1.97	0.0148
18	Unknown	1.97	0.0132
22	Acetone	1.96	0.0128
41	2-Butanone	1.95	0.0125
48	2-Methylbutanal	1.91	0.0123
207	Unknown	1.91	0.0133
38	Ethyl acetate	1.89	0.0118
37	Nonane	1.89	0.0119
107	2-Ethyl-5-methylfuran	1.89	0.0125
294	4-Methyl-3-pentenoic acid	1.86	0.0124
13	(E)-2-Methyl-1,3-pentadiene	1.86	0.0107
62	Benzene	1.84	0.0135
36	Unknown	1.79	0.0107
196	Unknown	1.78	0.0122
87	Unknown	1.77	0.0124
51	Unknown	1.74	0.0116
117	S-Methyl Thioacetate	1.74	0.0133
50	Unknown	1.73	0.0107
203	Thiazole	1.71	0.0127
129	Unknown	1.71	0.0115
55	(Z, Z)- 3,5-Octadiene	1.70	0.0106
7	Acetaldehyde	1.65	0.0119
49	2,4-Octadiene	1.64	0.0099
11	Dimethyl sulfide	1.62	0.0105
21	2-Methylpropanal	1.60	0.0120
170	Thiophene, 2-ethyl-	1.58	0.0137
64	2-Ethylfuran	1.58	0.0109
76	Decane	1.57	0.0115
315	Unknown	1.56	0.0114
299	Unknown	1.55	0.0121
300	2,6,10,14-Tetramethylpentadecane	1.54	0.0118

248	2-Nonanone	1.52	0.0116
93	4-Methyl-1,3-heptadiene	1.51	0.0101
56	Unknown	1.51	0.0098
42	Unknown	1.49	0.0128
59	Unknown	1.49	0.0096
122	2,3-Pentanedione	1.47	0.0103
79	Unknown	1.47	0.0098
78	Unknown	1.44	0.0110
313	Unknown	1.43	0.0112
15	TMA	1.42	0.0112
287	Unknown	1.37	0.0091
295	Undecanal	1.35	0.0100
337	Ethyl hexadecanoate	1.35	0.0075
131	Unknown	1.35	0.0107
242	Unknown	1.35	0.0074
57	Unknown	1.33	0.0073
336	Unknown	1.31	0.0102
174	Heptanal	1.31	0.0091
115	(E, E)- 1,3,6-Octatriene	1.29	0.0110
298	Cyclohexanol, 1-ethynyl-	1.29	0.0126
125	Butylcyclohexane	1.28	0.0084
324	Nonadecane	1.27	0.0069
1	Unknown	1.23	0.0079
251	Unknown	1.23	0.0079
32	2-Methylfuran	1.21	0.0065
116	Unknown	1.20	0.0095
168	Unknown	1.16	0.0059
138	Unknown	1.16	0.0070
194	4-Heptenal	1.13	0.0096
98	Unknown	1.13	0.0071
329	Ethyl tetradecanoate	1.10	0.0066
217	Cyclohexanone	1.09	0.0102
128	Undecane	1.09	0.0020
139	3-Pentanol	1.08	0.0091
28	Unknown	1.07	0.0105
204	Unknown	1.06	0.0086
148	Ethyl benzene	1.05	0.0045
319	Octadecane	1.04	0.0075
72	3-Pentanone	1.04	0.0089
71	Unknown	1.02	0.0032
193	Unknown	1.00	0.0041
234	2-Methyl-3-pentanol	1.00	0.0068

Table S7. Calibration curves and R^2 values for the quantification of flavor candidates in SO and CO.

No.	Compounds name	SO			CO		
		slope	intercept	R^2	slope	intercept	R^2
7	Acetaldehyde	973579	7691679	0.9912	529368	5095731	0.9905
11	Dimethyl sulfide	8130700	262220	0.9982	5626078	10953	1.0000
15	TMA	1.08	0.28	0.9793	1.08	0.28	0.9793
17	Propanal	1941383	39668659	0.9924	1191519	21885277	0.9942

21	2-Methyl propanal	5272849	155066	0.9998	2871916	101347	1.0000
72	3-Pentanone	120881771	4045533	0.9998	58589644	4913441	0.9996
109	1-Propanol	2459004	1043187	0.9801	2173202	683449	0.9901
117	S-Methyl thioacetate	89010644	58138	0.9995	25371696	15424	0.9994
122	2,3-Pentanedione	4376901	23799759	0.9999	2414467	13574979	0.9918
217	Cyclohexanone	27124319	77759	0.9929	7252340	11644	0.9997
238	*trans* -3-Hexen-1-ol	32489319	375320	0.9990	15320631	121063	0.9999
243	*cis* - 3-Hexen-1-ol	55032473	742633	1.0000	20778886	291705	0.9993
248	2-Nonanone	171951540	4958841	0.9995	37807120	87841	0.9995
267	1-Heptanol	79583541	6230607	0.9970	32170307	724445	0.9984
277	*trans, trans*-2,4-Heptadienal	623932	1792999	0.9765	458367	1047157	1.0000
300	2,6,10,14-Tetramethylpentadecane	1109748	103029477	0.9996	170582	376339	0.9968

Table S8. Lipid contents in each sample.

Sample name	%
RB	4.0
CH	4.2
SO	9.1
RA	19.3
CO	28.3

Values are expressed as means of duplicate measurements (n = 2). Lipids were extracted using the Folch method.
